# Integrated Electrocoagulation, Ultrafiltration, Membrane Distillation, and Crystallization for Treating Produced Water

**DOI:** 10.3390/membranes13060597

**Published:** 2023-06-12

**Authors:** Mahmood Jebur, Yelyzaveta Bachynska, Xiaolei Hao, Sumith Ranil Wickramasinghe

**Affiliations:** 1 Ralph E. Martin Department of Chemical Engineering, University of Arkansas, Fayetteville, AR 72701, USA; mgjebur@uark.edu (M.J.); bachynska.yelyzaveta@gmail.com (Y.B.); 2 Department of Chemical Engineering, Tikrit University, Tikrit 34001, Iraq; 3 Department of Biomedical Engineering, University of Arkansas, Fayetteville, AR 72701, USA; xh009@uark.edu

**Keywords:** water recovery, membrane distillation, scale formation, pretreatment, electrocoagulation

## Abstract

Produced water (PW) generated from hydraulic fracturing operations was treated using an integrated electrocoagulation, ultrafiltration, membrane distillation, and crystallization process (EC UF MDC). The aim was to determine the viability of this integrated process for maximizing water recovery. The results obtained here indicate that optimizing the various unit operations could lead to increased recovery of PW. Membrane fouling limits all membrane separation processes. A pretreatment step to suppress fouling is essential. Here, removal of total suspended solids (TSS) and total organic carbon (TOC) was achieved by electrocoagulation (EC) followed by ultrafiltration (UF). The hydrophobic membrane used in membrane distillation may be fouled by dissolved organic compounds. Reducing membrane fouling is essential to increase the long-term durability of the membrane distillation (MD) system. In addition, combining membrane distillation with crystallization (MDC) can help reduce scale formation. By inducing crystallization in the feed tank, scale formation on the MD membrane was suppressed. The integrated EC UF MDC process can impact Water Resources/Oil & Gas Companies. Conservation of surface and groundwater is possible by treating and reusing PW. Additionally, treating PW reduces the amount of PW disposed in Class II disposal wells and promotes more environmentally sustainable operations.

## 1. Introduction

The fastest growing energy sector in the US is unconventional shale gas and oil production. Hydraulic fracturing combined with horizontal drilling is used for exploitation of tight rock formations containing abundant oil and gas resources that were previously unreachable [1]. The extraction of shale gas using advanced hydraulic fracturing has increased from 14% of U.S. natural gas production in 2004 to 97% in 2018. However, this increase has led to a concurrent increase in water usage [2,3]. In hydraulic fracturing, water, mixed with chemicals, is pumped at high pressure through the well bore to fracture the tight rock formation. Subsequently, the pressure is reduced, and the water flows back to the surface as flowback and produced water, known collectively as PW. Around 15–23 million liters of PW are generated during the extraction period of each well [4]. Approximately 116 billion liters of PW are produced in the U.S. annually [5]. About 20.06 million liters of water were used per well in the Fayetteville shale [6].

Water is a very scarce and valuable natural resource. Promoting circularity in water usage is essential in order to develop sustainable manufacturing processes. Recovery and reuse of PW are essential. The work conducted here directly addresses this major societal issue. PW is highly impaired; thus, treating this water is very challenging. It contains a range of contaminants, as well as high total dissolved solids (TDS) concentration, high total suspended solids (TSS), polar and non-polar organic compounds, and low surface tension dissolved species [7,8,9,10]. Currently, the PW is frequently deep well injected. However, deep well injection practices are non-sustainable [11] and have several drawbacks, including the limitation of available deep well injection sites, the cost of transporting PW to the available sites, and the possibility of creating earthquakes. Importantly it does not lead to the recycling and reuse of water. 

Limited options exist to treat PW. Some investigators have considered distillation-based technologies, such as multistage flash distillation, mechanical vapor recompression, or integrating evaporation, crystallization, and spray drying to treat PW [12]. Though successful in treating high TDS brines, these technologies suffer from some drawbacks, such as high cost, large footprint, and the use of chemicals [13]. Here, membrane technology is considered to treat high TDS brines. 

Conventional membrane processes such as reverse osmosis (RO) can be used to treat brines with TDS values below 50,000 mg/L [14]. However, as the osmotic back pressure increases, the amount of water that can be recovered using RO, especially as the TDS increases over 50,000 mg/L, is limited [14,15]. Membrane distillation (MD) is an emerging technology that can be used to treat high TDS PW. In MD, a microporous hydrophobic membrane is used as a barrier between the feed and permeate streams. The feed stream is heated relative to the permeate stream. This imposed temperature gradient leads to a vapor pressure gradient across the membrane. Water vapor passes down the vapor pressure gradient from the feed to the distillate. Importantly non-volatile solutes cannot pass through membrane pores. Unlike reverse osmosis, the depression of the feed vapor pressure with increasing feed TDS is much less than the increase in osmotic back pressure [16,17]. Here, direct contact membrane distillation is used where the feed and permeate streams are in direct contact with the two surfaces of the membrane [18].

However, like all other membrane technologies, MD suffers from fouling of the membrane by rejected species. In the case of hydraulic fracturing PW, dissolved polar and non-polar organic species can easily adsorb onto the hydrophobic membrane surface. In addition, low surface energy compounds, such as surfactants, can adsorb onto the membrane. Scaling by dissolved salts at high TDS can also occur. Fouling leads to a drop in permeate flux. However, it can also lead to failure of the membrane whereby water and dissolved non-volatile solutes pass directly through the membrane pores [19]. 

Our aim is to develop an integrated process to maximize water recovery and suppress membrane fouling. Commercially available polyvinylidene fluoride (PVDF) membranes have been used to evaluate the feasibility of the integrated electrocoagulation ultrafiltration membrane distillation crystallization (EC/UF MDC) process to address both scaling and wetting of the membrane and to maximize water recovery. Electrocoagulation (EC) is used to pretreat the feed and remove dissolved organic compounds that could foul the membrane. UF is used to rapidly separate the permeate from the EC sludge. MD is used to recover treated water. By linking this with crystallization, we maximize water recovery while suppressing scaling on the MD membrane. 

EC is an electrolysis process where a sacrificial electrode (anode) is used to generate metal ions. These metal ions generate a variety of metal hydroxides as follows: M_(s)_ → M^n+^_(aq)_ + ne^−^ at the anode. Water is reduced at the cathode by the reaction 2H_2_O + 2e^−^ → 2OH^−^ + H_2_, where M is often Al or Fe [20]. Metal complexes such as M(OH)^(n−1)+^, M(OH)_2_^(n−2)+^ and M_6_(OH)_15_ ^(6n−15)+^ are produced. These metal complexes contribute to the neutralization of the negatively charged organic species and suspended solids. As the solution ages, they convert to amorphous M(OH)_n(s)_ particles. M(OH)_n(s)_ particles can easily adsorb and trap organic compounds and suspended solids. EC can effectively remove a wide range of contaminants, including suspended solids, emulsified oils, heavy metals, and some organic compounds. Using EC as a pretreatment step can provide several benefits, including the high removal of contaminants, operational flexibility, production of 80% fewer solids, no hazardous waste disposal cost, and reduced use of expensive chemical agents.

**Figure 1:**
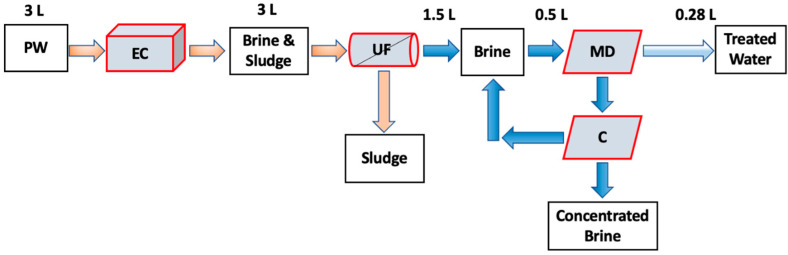
Schematic diagram of the EC UF MDC process studied here. C refers to the crystallization unit.

Crystallization after EC UF MD can minimize membrane fouling and scaling by reducing the formation of crystal nuclei in the bulk feed. This is particularly important when using hypersaline PW with a high TDS concentration. In addition, the EC UF MDC process can also offer a potential solution to high TDS brine disposal by recovering both water and minerals, which can lead to a nearly zero liquid discharge [21,22]. Here, the feasibility of using EC UF MDC to recover water and minerals from hypersaline shale gas PW has been investigated. In addition, we compared the increase in water recovery when adding a crystallization unit by comparing EC UF MDC with EC UF MD. As about 80% of the water utilized in hydraulic fracturing is surface water and groundwater, the process developed here could have an impact on hydraulic fracturing operations [23]. Further, about 95% of the collected PW is directly disposed of in a Class II disposal well [24]. Figure 1 shows the concept of the combined processes. The feed consisted of 3 L. After UF, about 1.5 L of permeate is recovered. Table 1 shows previous studies on treating synthetic and actual wastewater, including PW, seawater, and RO brines. Previous studies have been conducted using EC MD or MDC systems to treat low TDS brines as well as synthetic PW. To our knowledge, this is the first study that has considered an integrated EC UF MDC system to treat hypersaline PW from unconventional oil and gas wells. 

## 2. Methods

### 2.1. PW Characterization 

PW samples were collected from a hydraulic fracturing facility in Midland, Texas, USA. The samples were analyzed at the Arkansas Water Resources Center, University of Arkansas (Fayetteville, AR, USA). The received PW had been treated with chlorine dioxide at the hydraulic fracturing facility to remove bacteria and iron. EPA standard methods 160.1, 160.2, 415.1, and 180.1 were used to measure TDS, TSS, turbidity, and total organic carbon (TOC) [31]. EPA methods 200.7 and 300.0 were used to measure cations and anions, respectively. Conductivity was measured using a conductivity meter (VWR, Radnor, PA, USA). 

### 2.2. Membrane Characterization

Static water contact angles were measured using a sessile drop contact angle goniometer (Model 100, Rame-Hart Instrument Company, Netcong, NJ, USA). The DI water droplet volume was 3 µL introduced at a rate of 0.5 µL/s. Each droplet was allowed to stabilize for 10 sec prior to measurement. For each membrane, the average value of three measurements obtained at three different locations was used in this study. 

For each membrane before and after MD or MDC, both the surface morphology and elemental analysis were obtained using scanning electron microscopy (SEM) and energy-dispersive X-ray (EDX) spectroscopy, respectively, using Nova Nanolab 200 Duo-Beam Workstation (FEI, Hillsboro, OR, USA). 

### 2.3. EC UF Pretreatment

**Figure 2:**
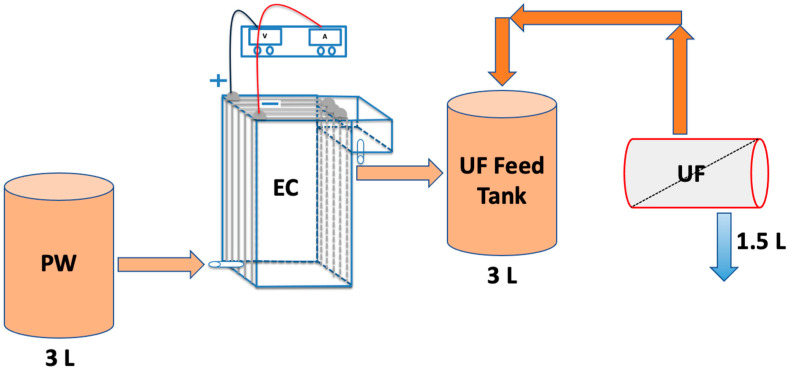
Schematic representation of the EC UF system investigated here.

Figure 2 shows the EC UF system. Here, a custom-built polycarbonate reactor with a volume of 1078 cm^3^ (dimensions of 7 cm × 11 cm × 14 cm) was used to conduct all EC experiments. Five aluminum electrodes were fitted vertically inside the reactor. The inter-electrode spacing was 10 mm. The residence time in the reactor was 5 min. A DC power supply (Hewlett Packard, Palo Alto, CA, USA) was connected to a reverse polarity switch which enabled the direction of the current to alternate every 30 s. This step is essential, as shown by our earlier work, to prevent the formation of a passivation layer on the electrode surface which would suppress further reactions [32,33]. 

The first and last electrodes were connected to the power supply in a bipolar series (BPS) configuration to simplify the electrical connections. In previous studies, the BPS configuration was shown to lead to an enhancement in TOC reduction [34]. In earlier studies, several EC experiments were conducted to determine the appropriate current and reaction time [34,35]. Based on these earlier studies, a range of currents from 1 to 3 A and a reaction time of 5 min were studied here. 

EC is an electrolysis process where aluminum ions are continuously generated at the anode while the reduction of water takes place at the cathode leading to the formation of hydrogen gas and hydroxide ions [34,35]. However, the actual reactions that occur depend on the reduction potentials of the other species present in the feed. A range of poly aluminum hydroxides is produced in the solution when coagulating ions (aluminum and/or hydroxide ions) undergo hydrolysis in water. These aluminum hydroxides can help destabilize suspended, emulsified, and dissolved contaminants which can aggregate and precipitate as sludge or lift up to the surface as flocs. 

Firstly, three standalone EC experiments were conducted to optimize the current based on achieving a high TOC reduction. After each EC experiment, treated water was removed from the sludge and settled floc after sedimentation for 30 min. After optimizing the current, the EC reactor was run to treat 3 L of PW. This process takes about 15 min, which leads to an average floc aging time of 7 min. Next, the supernatant from the EC becomes the feed to the UF process. 

The UF process was conducted after EC using a ceramic membrane from CeraMem (Waltham. MA, USA). Crossflow filtration was conducted using a 10 nm nominal pore-size ceramic membrane module. The active surface area was 0.13 m^2^. After EC, the 3 L of PW was placed in the UF feed tank. The feed was recirculated through the module using a diaphragm pump (P800, King-Kong, Triwin, Taichung City, Taiwan), keeping the permeate outlet closed. The permeate outlet was opened after 5 min. The transmembrane pressure (TMP) was 65 kPa at a feed flow rate of 2.5 L/min. The permeate water was collected in the permeate tank, which was placed on a balance (Mettler Toledo, Columbus, OH, USA). The permeate flux was calculated based on the weight of permeate. About 50% of the EC-treated water was collected as permeate. After each experiment, the membrane was cleaned by pumping hot DI water for 1 h prior to starting a new experiment. 

### 2.4. MD Testing

**Figure 3:**
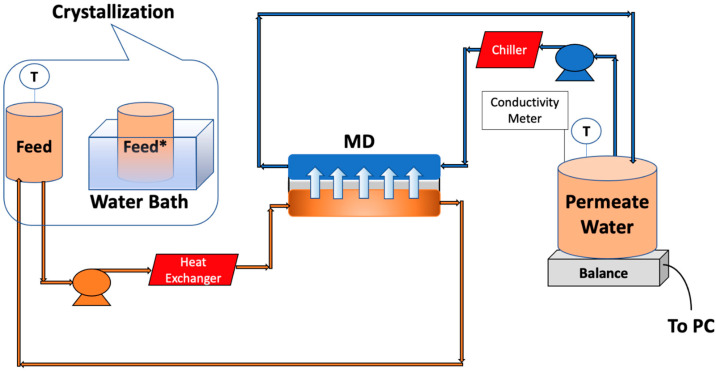
Schematic representation of MD setup along with crystallization. * The feed was placed in the water bath after recovering 10% of the water.

The MD system used here is shown schematically in Figure 3. A custom-made acrylic module was used. The total membrane surface area was 40 cm^2^. The flow channel was 2 mm deep. A commercial 0.65 μm pore size PVDF membrane (Millipore, Billerica, MA, USA) was used. PTFE spacers were also used (ET 8700, Industrial Netting, Minneapolis, MN, USA) to provide mechanical support and promote mixing. Peristaltic pumps (Masterflex I/P, Cole Parmer, Vernon Hills, IL, USA) were utilized to pump the feed and permeate streams at 0.5 L/min on opposite sides of the membrane. The temperature of the permeate and feed tanks was maintained at 20 °C and 60 °C using an external chiller and heater, respectively (PolyScience, Niles, IL, USA). Experiments were run for about 8 h. We aimed for about 40% water recovery. 

### 2.5. MDC Testing

MDC experiments were conducted in four stages. Initially, MD was run till about 10% of the feed was recovered in the permeate (about 115 min operation). The feed tank was then placed in a water bath containing ice that was constantly replaced. After about 15 min, the temperature of the feed reached 20 °C. It was then kept in the water bath for an additional 5 min. After removal of the precipitate, the feed tank was returned to the MD system, and MD recommenced once the temperature reached 60 °C. The precipitate was recovered and analyzed for the feasibility of mineral recovery. Precipitation occurs in the feed tank rather than on the membrane, increasing water recovery. 

Using the weight change of the permeate tank, the water flux was calculated and normalized using the average flux during the first 15 min of operation. The permeate conductivity was measured using a conductivity meter (VWR, Radnor, PA, USA). Each MD and MDC experiment was conducted using 500 mL of PW with no pretreatment or with PW pretreated using EC UF. 

An experiment was also conducted where the membrane was regenerated and reused. A membrane regeneration cycle was applied once 40% of the feed volume was recovered or there was no permeate weight increase for 20 min. During regeneration, DI water was pumped on both sides of the membrane at 0.5 L/min for 1 h. 

## 3. Results and Discussion 

### 3.1. Wastewater Characterization 

Table 2 provides the water quality parameters of the PW received from the hydraulic fracturing facility, as well as after EC UF. The TDS is about four times higher than seawater. The major inorganic species present are chlorine (83,117 mg/L), sulfate (545 nmg/L), calcium (2396 mg/L), magnesium(383 mg/L), potassium (1089 mg/L), and sodium (55,902 mg/L). A high concentration of calcium ions could lead to membrane scaling due to the precipitation of calcium sulfate [36]. The TOC and TSS are also high, about 395 mg/L and 187 mg/L, respectively. The quality of the PW, in general, is highly variable, affecting the treatment operations’ efficiency. As can be seen, EC UF pretreatment leads to a 94% decrease in TOC and a 59% decrease in TSS. A corresponding decrease in turbidity is also observed. However, as expected, EC does not significantly change the concentration of dissolved ions.

### 3.2. Membrane Characterization

**Figure 4:**
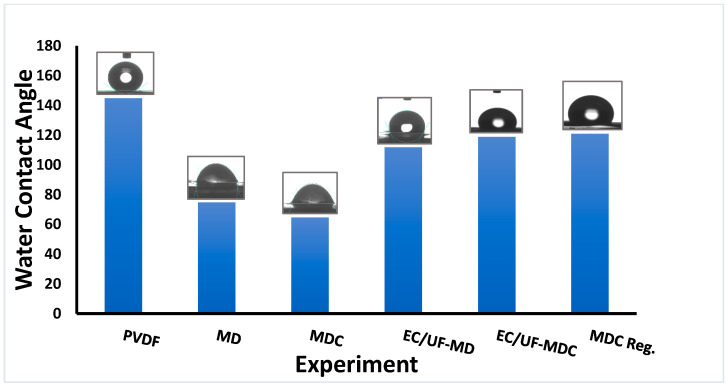
Water contact angle measurements. PVDF refers to virgin membrane; MD and MDC refer to PVDF membranes after MD and MDC, respectively, whereas EC/UF-MD and EC/UF-MDC refer to the same unit operations where the feed is pretreated using EC/UF; MDC Reg. is for the membrane after regeneration.

The water contact angles are given in Figure 4 before and after MD and MDC. Initially, the PVDF membrane is hydrophobic as the water contact angle is 145°. It is important for MD operation that water vapor and not water should pass through the membrane pores. Figure 4 indicates that during MD, the water contact angle decreases. This change is due to the adsorption of dissolved organic compounds on the membrane surface. The water contact angle is 75° after MD and 65° after MDC in the absence of pretreatment. This contact angle difference could enhance scale deposition on the layer of adsorbed organic compounds, especially if they are polar [37]. Thus, reducing the TOC and TSS by EC UF is important to reduce membrane fouling. As shown in Figure 4, when the PW is pretreated with EC UF after MD and MDC, the contact angle is 112° and 119°, respectively. This difference is significantly higher than in the absence of pretreatment.

**Figure 5:**
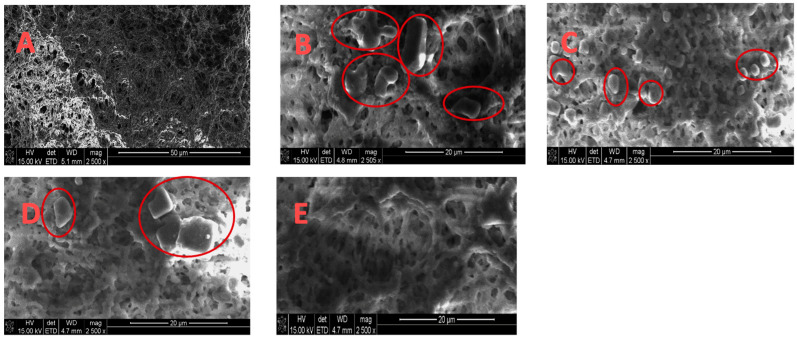
SEM images (same magnification) for (**A**) virgin membrane; (**B**) after MD; (**C**) after MDC; (**D**) after MD using EC UF pretreated PW; (**E**) after MDC using EC UF pretreated PW.

Figure 5 gives corresponding SEM images of the membranes before and after MD and MDC. The SEM images were captured at magnifications of 2500×. The feed flow rate was 0.5 L/min, which results in a Reynolds number of 200 (laminar flow). The SEM images of the membranes before MD as well as after MD and MDC, and in the absence and presence of pretreatment, are given in Figure 5A–E, respectively. As can be seen, some deposition (highlighted with red circles) on the membrane surface is observed after MD, while very minimal deposition is observed after MDC and specifically MDC with pretreated feed PW. Thus, pretreatment using EC UF, followed by MD and then crystallization, appears to suppress fouling on the membrane surface.

Results for elemental analysis of the membranes using EDX are given in Table 3. The average elemental ratios of carbon/fluorine (C/F) and oxygen/fluorine (O/F) for the PVDF membranes after MD and MDC are given in Table 3. As can be seen, the C/F and O/F ratios are high for PVDF membranes after MD and MDC in the absence of pretreatment, which is mainly due to the organic fouling. The contact angle, SEM, and elemental analysis results indicate that reducing both TOC and TSS by EC UF leads to a significant decrease in deposition of colloidal material and organic compound on the membrane surface. The results highlight the importance of using an integrated pretreatment step to minimize membrane fouling when treating highly impaired wastewaters, such as hydraulic fracturing PW. 

### 3.3. EC Performance

The aggregated aluminum hydroxides produced during EC will adsorb soluble organic compounds. The low-density flocs rise to the surface, age, densify, and sediment. This adsorption phenomenon is a result of the liquid-solid intermolecular attractive forces between the organic solutes in solution and the large surface area of the porous flocs that form. TOC reduction is calculated using Equation (1)

(1) $$TOC\%=\left(\left(X_{pw}-X_{rw}\right)/X_{pw}\right)*100$$

where *X_pw_* and *X_rw_* are the TOC in the *PW* and the treated water after EC, respectively. 

Table 4 gives the TOC concentration and its reduction percent for different currents. TOC reduction increases from 65% to 74% as the current increases from 1 to 3 A. To obtain a higher reduction of TOC, higher currents and longer reaction times are needed [34,35]. However, it is also important to ensure that the EC process is practical. Based on our earlier studies [34], long reaction times are undesirable as they lead to holding very large volumes of water, thus, a rapid increase in the capital cost for the process and an increased footprint. Based on the higher TOC reduction, a current of 3 A is used here. Reduction of TOC by EC is essential in order to minimize the adsorption of organic species that will lead to fouling of the membrane and reduced fluxes. EC reduces the load of organic compounds, including surfactants and polar organic compounds, in the feed stream. Surfactants, in particular, can deposit on the hydrophobic membrane. Their more hydrophilic domain remains exposed to the feed stream, which can enable scale formation on the deposited organic compounds. This situation is particularly serious when working with hypersaline solutions and, in turn, can increase the likelihood of water passing through the membrane pores. The membrane surface will be much more hydrophilic, leading to the failure of the process. 

### 3.4. UF Performance

While sedimentation may be used to recover the treated water from the sludge, it is unlikely to be practical. Long sedimentation times will lead to the need for very large holding tanks and, therefore, higher capital costs and footprint. Further, given the high variability in the quality of the PW, even from the same well, the sedimentation time is likely to be highly variable [35]. Here, UF is used to rapidly remove the EC sludge from the supernatant. It should be noted that floc aging is necessary in order to allow the coagulation processes to occur, whereby dissolved organic compounds and colloids are adsorbed onto the growing aluminum hydroxide flocs. Here, the average floc aging time is about 7 min. In a continuous process, a holding tank will be required after the EC unit to allow the floc to age. 

**Figure 6:**
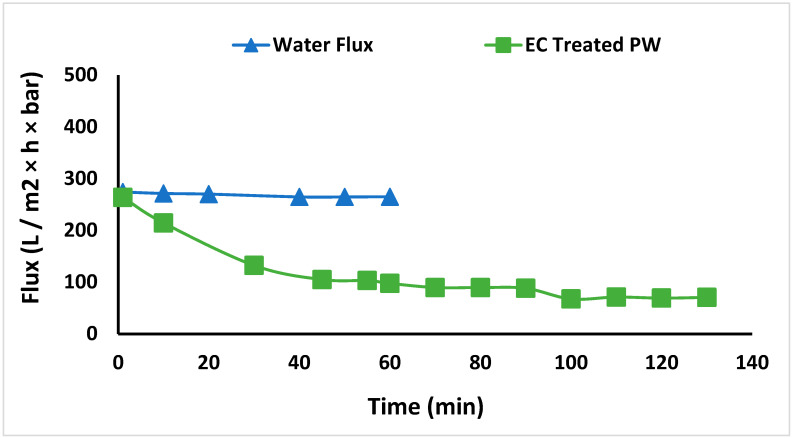
DI water flux and flux of permeate stream after EC.

EC was conducted on the entire 3 L feed, after which UF was conducted at a TMP of 65 kPa. The variation of permeate flux with operating time is shown in Figure 6. First, the membrane was tested with DI water to determine the initial DI water flux of 270 L m^−2^ h^−1^ bar^−1^. Then, the membrane was used to treat EC-pretreated PW using constant pressure filtration. The flux gradually decreased to 71 L m^−2^ h^−1^ bar^−1^ and stabilized at 70 L m^−2^ h^−1^ bar^−1^ even after 100 min. The decrease in flux with time is due to the deposition of flocs on the membrane surface. The membrane was regenerated after 50% recovery of the feed water by simply recirculating the hot water for 1 h. The DI water flux was determined again. The DI water flux is similar to the flux during the first run (within 5%). The result suggests that EC was effective at flocculating the dissolved organic compounds and particulate matter that could plug the membrane pores. Further, regenerating the UF membrane by circulating hot water is sufficient to remove flocs from the membrane surface, resulting in minimal irreversible fouling. 

### 3.5. MD and MDC Performance

**Figure 7:**
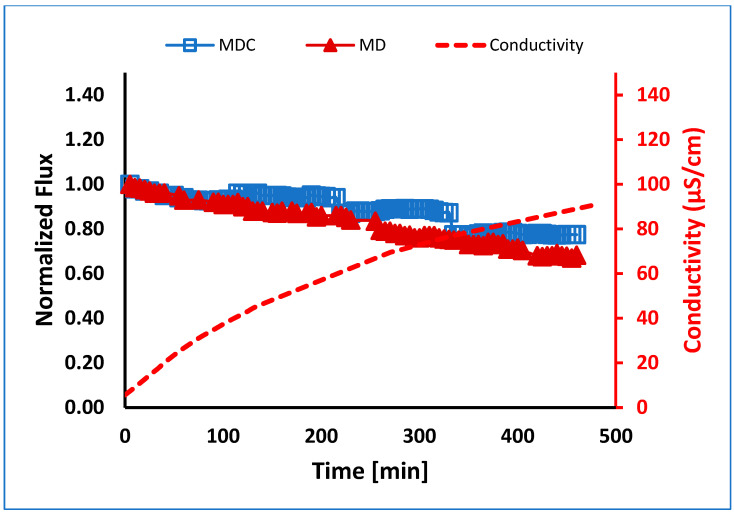
Normalized flux versus time for the membrane using MD and MDC for PW at 0.5 L/min flow. MDC was conducted in four stages, each running for about 115 min.

Normalized flux versus time for MD and MDC of PW in the absence of pretreatment is shown in Figure 7. Total water recovery is shown in Table 5. As can be seen, the flux for MDC shows four slight jumps due to the mode of operation used here. After 10% of the feed volume was recovered as permeate, MD was stopped, and the feed was cooled to promote precipitation, as described in the methods section. The jump in permeate flux occurs when MD recommences. However, as can be seen in Figure 7, the permeate flux and water recovery are higher for MD integrated with crystallization. The conductivity of the permeate samples collected from MD and MDC experiments gradually increased. Values for both MD and MDC were within 5% of each other. The average value is shown in Figure 7. The maximum value reached was about 90 μS/cm due to the passage of volatile inorganic compounds, such as ammonium chloride, from the feed stream to the permeate stream [38]. The concentration of ammonium ions in the permeate samples was in the range of 10 to 18 mg/L. Table 5 summarizes the volume of feed water recovered. As can be seen, water recovery is significantly enhanced using MDC. Here, the use of MDC reduced the risk of supersaturation and precipitation on the membrane surface. 

**Figure 8:**
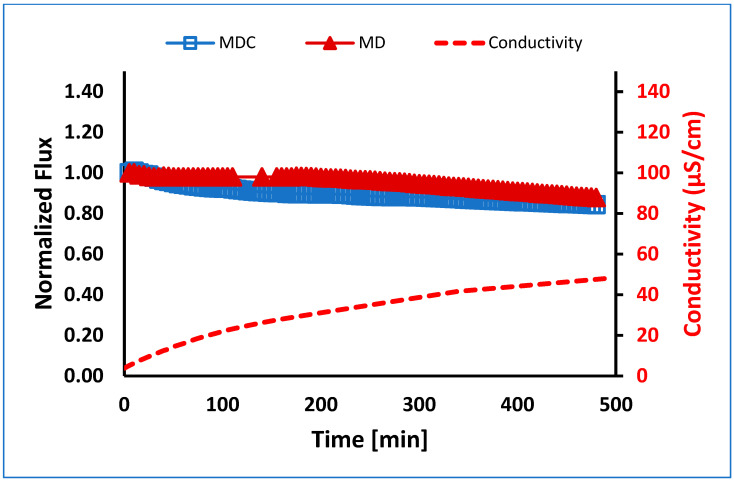
Normalized flux versus time for MD and MDC for EC UF pretreated PW at 0.5 L/min flow. Note: MDC was conducted in four stages, each running for about 115 min.

Crystallization showed no significant differences in membrane performance when comparing MDC and MD for EC UF pretreated PW (see Figure 8). This result is probably due to first reducing the layer of adsorbed organic species, which further decreases the likelihood of precipitation of dissolved salts on the membrane surface. The conductivity for both MD and MDC experiments increased with values within 5% of each other. 

**Figure 9:**
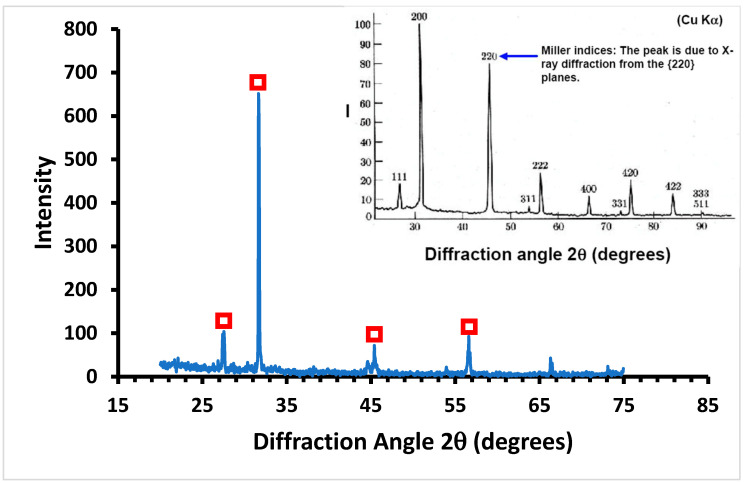
XRD analysis of the salts produced during MDC. The XRD spectrum of NaCl (standard) is shown in the inset. Red boxes denote the main peaks in the XRD analysis of the sample assigned to the XRD spectrum of NaCl standard.

An average salt recovery of 42 kg/m^3^ was obtained after cooling the feed to 20 °C for 5 min. X-ray diffraction (XRD) was used to identify the purity of the salts produced. Figure 9 shows the XRD patterns of the crystals produced during MDC. The results indicate that the main salts formed are halite (sodium chloride), a monovalent ion of low crystallinity, as shown in Figure 9. 

**Figure 10:**
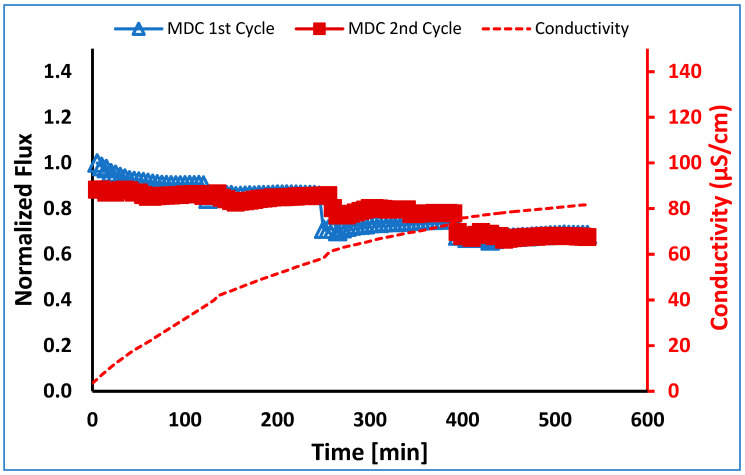
Normalized flux versus time for MDC of a PW feed stream. The membrane was regenerated and run a second time. Note: MDC first cycle run till 40% water recovery after membrane regeneration; the second cycle run till 40% water recovery.

Finally, in order to determine if membrane fouling is reversible and to determine the feasibility of membrane regeneration, the membrane was regenerated after 40% of the feed volume was recovered and then run with a new batch of 500 mL feed. As can be seen in Table 5, during the second run, 40% water recovery was achieved. The normalized fluxes for the two runs were within 10% of each other, as shown in Figure 10. The membrane could be easily regenerated simply by pumping water on both sides of the membrane for 1 h. Importantly, the conductivity values for both runs were within 5% of each other. The average values are given in Figure 10.

In this work, we used a commercially available hydrophobic membrane that is not designed for MD. Today there are no membranes specifically designed for MD applications. Consequently, the long stability of the membranes remains an important issue. The development of commercial MD processes will be enabled by the development of robust commercial MD membranes. 

Taken together, the results obtained here suggest that EC UF MDC could be a viable process to treat hydraulic fracturing PW. Our earlier studies [35] indicate that the water recovered after MD is suitable for discharge. It should be noted that while the addition of a crystallization step will drive the process toward zero liquid discharge, it adds significantly to the cost of the process. Operation of the process without a crystallization step will still lead to tremendous water savings, minimize the need for deep well injection, and lead to a more sustainable process. However, it is important to realize that dissolved volatile species can pass through the membrane during MD. Consequently, the recovered water will contain these species. Thus, the quality of the recovered water will depend on the feed water quality. This work highlights the importance of integrating unit operations when developing wastewater treatment strategies. 

In order to optimize the process, further studies should be conducted using a continuous process, helping to determine the required floc aging time and the maximum feasible water recovery using MDC. It is essential that membrane wetting be avoided where water passes directly from the feed to the permeate. Thus, a practical upper limit for water recovery exists. Similarly, membrane regeneration should be conducted such that irreversible fouling is avoided. It is important that regeneration leads to flux recovery. In addition, MDC could lead to sludge mining, which, in turn, may provide a valuable byproduct that could help offset the cost of water treatment. 

## 4. Conclusions

This investigation is one of the first studies to investigate the use of EC UF MDC for treating hypersaline hydraulic fracturing PW. The combined EC UF MDC process was used to treat hydraulic fracturing PW. The PW manifested a high TDS, TSS, and TOC. Nevertheless, 40% of the feed volume was recovered. It is likely that greater water recovery is possible. By using crystallization after MD, precipitation on the membrane is suppressed. Adequate reduction in the PW TOC can be achieved using EC. UF is then used to efficiently remove the particulate matter produced during EC. The stability of the membrane is critical. Here, a commercially available PVDF membrane was used. The membrane was robust and easily regenerated. 

The EC UF MDC technology can have an impact on water resources and oil and gas companies, as surface and groundwater form about 80% of the water used in hydraulic fracturing. The process developed here could be used to treat and reuse PW. If a crystallization step is not included, the operational cost of the process will be reduced while still providing significant water recovery. The possibility of mining the precipitate from the crystallization tank could lead to valuable byproducts that could help offset the cost of water treatment. The data collected from treating PW can be used to evaluate the integrated EC UF MDC process, which can guide further development of the process. 

## Figures and Tables

**Table 1:** Overview of the previous studies available in the current literature.

Treatment Configuration	Feed Water	TDS (mg/L)	Reference
MDC	Actual RO Brines	50,000	[25]
MDC	Synthetic PW	150,000	[26]
MDC	Actual PW	30,000	[27]
MDC	Synthetic RO Brines	65,000	[28]
Ultrasound-Assisted MDC	Synthetic NaCl Solution	350,000	[29]
MDC	Actual RO Brines	45,000	[30]

**Table 2:** Water quality for PW as received and after EC UF pretreatment operation.

Parameter	Unit	PW *	EC/UF PW **
TDS	mg/L	137,247	121,037
TOC	mg/L	395	23.3
TSS	mg/L	187	76.4
Turbidity	NTU’s	147	0.6
pH	-----	7.4	7.3
Conductivity	µS/cm	166,300	312,000

* PW as received; ** PW after EC UF pretreatment.

**Table 3:** The C/F and O/F atomic percent ratios for PVDF membranes.

Membranes	C/F Atom Percental Ratio	O/F Atom Percental Ratio
After MD (no pretreatment)	2.4	0.37
After MDC (no pretreatment)	1.8	0.23
After EC/UF-MD	1.7	0.19
After EC/UF-MDC	1.6	0.12

**Table 4:** TOC reduction at different currents (1, 2, and 3), 5 min reaction time, and using 5 Al electrodes in BPS configuration.

Current (A)	TOC (ppm)	Reduction of TOC (%)
1	140	65
2	124	68
3	102	74

**Table 5:** Summary of water and salt recovery after MD and MDC at 0.5 L/min flow.

Experiment	Water Recovery (mL)
MD of PW	175
MDC of PW	205
MD of EC/UF Pretreated PW	220
MDC of EC/UF Pretreated PW	215
MDC of PW with regeneration (1st Cycle)	200
MDC of PW with regeneration (2nd Cycle)	201

## Data Availability

No new data were created.

## Abbreviations

BPS	Bipolar Series
C/F	Carbon/Fluorine
EC	Electrocoagulation
EDX	Energy-dispersive X-ray
MD	Membrane Distillation
O/F	Oxygen/Fluorine
PVDF	Polyvinylidene Fluoride
PW	Produced Water
RO	Reverse Osmosis
SEM	Scanning Electron Microscopy
TDS	Total Dissolved Solids
TMP	Transmembrane Pressure
TOC	Total Organic Carbon
TSS	Total Suspended Solids
XRD	X-ray Diffraction
Equation Symbols:	
TOC%	Reduction of Total Organic Carbon
*X_pw_*	Total Organic Carbon in Produced Water
*X_rw_*	Total Organic Carbon in Treated Water After Electrocoagulation
